# Yifei Decoction Regulates the NGF/TRKA/PI3K/AKT Signaling Axis to Inhibit the Epithelial–Mesenchymal Transformation and Proliferation of Pulmonary Epithelial Cells in Bleomycin‐Induced Pulmonary Fibrogenesis

**DOI:** 10.1155/carj/6614209

**Published:** 2026-01-30

**Authors:** Lijuan Chen, Chengzhong Lan, Xiangli Deng, Mei Wu, Mei Shao, Mei Yang, Xinwen Ma, Fen Huang, Haiyin Wu

**Affiliations:** ^1^ Department of Pulmonary Diseases, The First Affiliated Hospital of Yunnan University of Chinese Medicine, Yunnan Provincial Hospital of Traditional Chinese Medicine, Kunming, 650000, Yunnan, China, yn-tcm-hospital.com

**Keywords:** EMT, idiopathic pulmonary fibrosis, NGF/TRKA/PI3K/AKT signaling pathway, Yifei decoction

## Abstract

**Background:**

It was important to find better therapeutic drugs for idiopathic pulmonary fibrosis (IPF), and in our previous study, Yifei decoction (YFT) alleviated IPF. A deeper understanding of its mechanisms of action could aid in the development of novel treatment strategies.

**Methods:**

We established an IPF mouse model by administering bleomycin (BLM), followed by treatment with YFT. Histopathological analysis of lung tissue was conducted to evaluate the effects of YFT, along with transcriptomic profiling to identify key regulatory molecules involved in its therapeutic action. Immunofluorescence staining was performed for TRKA and surfactant protein C (SP‐C) in IPF lung slices. A549 cells were induced with TGF‐β1 to assess the effect of YFT on alveolar epithelial cells, and cells overexpressing TRKA were constructed. Western blotting analysis was performed to detect EMT‐ and PI3K/AKT pathway‐related protein levels, and an EdU proliferation assay was conducted to measure the proliferation of A549 cells.

**Results:**

YFT intervention reduced pathological lung injury in BLM‐treated mice. GO enrichment analysis revealed enrichment of DEGs in the extracellular matrix and proteinaceous extracellular matrix. Analysis of the enriched KEGG pathways revealed enrichment of the PI3K–AKT signaling pathway. YFT also decreased the number of SP‐C^+^/TRKA^+^ cells in lung tissues, inhibited the expression of TRKA, and reduced the NGF concentration in TGF‐β1‐stimulated A549 cells. YFT reduced TRKA, PI3K, and AKT phosphorylation levels, EMT, and cell proliferation. However, these effects were eliminated when TRKA was overexpressed.

**Conclusion:**

YFT might regulate the NGF/TRKA/PI3K/AKT pathway to alleviate pulmonary fibrosis by reducing EMT and cell proliferation. This study laid the groundwork for future research on the possible enhancement of the therapeutic effect of YFT when YFT is combined with other medications.

## 1. Introduction

Idiopathic pulmonary fibrosis (IPF), a chronic disease, is a dysregulated response to alveolar epithelial injury [1]. The main feature of IPF is the formation of fibroblast foci, which are aggregates of proliferating fibroblasts and myofibroblasts within the mucoid matrix. This leads to changes in the cytoskeleton, extracellular matrix (ECM) components, and cell dysplasia, ultimately leading to lung scarring and destruction [2]. Fibroblasts arise from several mechanisms, including epithelial–mesenchymal transformation (EMT) [3], fibrocyte recruitment [4], pericellular transdifferentiation [5], pleural mesothelial cells [6], and local fibroblast population expansion [7]. About one‐third of fibroblasts in pulmonary fibrosis originate from epithelial cells [8]. Although many researchers have reported that the differentiation of circulating bone marrow‐derived progenitors and the transformation of resident fibroblasts are the sources of myofibroblasts, some studies have investigated the contribution of EMTs from alveolar epithelial cells. By undergoing EMT, alveolar epithelial cells can serve as a significant provider of fibroblasts and myofibroblasts [3]. Therefore, alveolar epithelial cells are required for the fibrotic development of IPF. After repeated injuries, the abnormal proliferation of alveolar epithelial cells leads to the production of multiple cytokines, particularly transforming growth factor (TGF)‐β1. This exacerbates epithelial cell injury, resulting in EMT [9]. The accumulation of myofibroblasts and fibroblasts, along with excessive deposition of collagen, is a significant outcome of the process known as EMT in type 2 lung epithelial cells and plays a key role in IPF [10]. Therefore, the abnormal proliferation and occurrence of EMT in alveolar epithelial cells are critical factors in IPF.

In recent years, the use of Chinese medicines to treat IPF has increased considerably, and these Chinese medicines have significant benefits, such as improvement of lung function and the quality of life [11]. The Chinese herbal medicine Yifei decoction (YFT) is composed of *Fructus Aurantii Immaturus* (aka Zhi shi in Chinese), *Semen Persicae* (aka Tao ren in Chinese), *Radix Platycodi* (aka Jie geng in Chinese), *Mori Cortex* (aka Sang bai pi in Chinese), *Rhizoma Atractylodis macrocephalae* (aka Bai zhu in Chinese), *Radix Salviae liguliobae* (aka Dan shen in Chinese), and *Glycyrrhizae Radix et Rhizoma* (aka Gan cao in Chinese) [12]. YFT can alleviate lung fibrosis in rats with phlegm‐heat obstructing lung syndrome [13]. It can also reduce inflammation in patients experiencing acute exacerbation of chronic obstructive pulmonary disease (COPD), accompanied by symptoms of qi deficiency of the spleen and lung [14]. In our previous study, when mitochondrial coenzyme Q (MitoQ) and YFT were combined, there was a decrease in the secretion of proinflammatory cytokines in rats with IPF [12]. However, the mechanism underlying the improvement of IPF by YFT requires further investigation. Therefore, in this study, we conducted RNA sequencing on lung tissues from bleomycin (BLM)‐induced mice that were treated with YFT, and we selected tropomyosin receptor kinase (TRKA) as a potential key molecule regulated by YFT for further investigation.

The TRK family comprises three tyrosine kinase receptors, which include TRKA, TRKB, and TRKC [15]. The involvement of TRKA in the onset and progression of different lung‐related illnesses, such as pulmonary sarcoidosis [16] and nonsmall cell lung cancer [17], has been demonstrated. Animal experiments have revealed that inhibiting TRKA may help prevent renal fibrosis [18]. Several studies have investigated the potential of TRKA inhibitors in treating various diseases. The cellular penetrating peptide IPTRK3 can bind to the enzyme‐active site of TRKA to simulate the intramolecular interaction of nonactive proteins. This interaction antagonizes the function of TRKA, thereby reducing neuropathic pain caused by sciatic nerve ligation [19]. Studies regarding TRKA in IPF are lacking. The activation of TRKA is primarily mediated by two tyrosine residues, Tyr490 (Y490) and Tyr785 (Y785), in the cytoplasmic domain [20]. After binding with nerve growth factor (NGF), phosphorylated TRKA recruits certain molecules and activates signaling pathways such as SHC–Ras–MAPK, phosphatidylinositol 3 kinase (PI3K)/protein kinase B (AKT), and protein kinase C (PKC), thereby regulating cell survival, proliferation, and differentiation [21–23]. Mammalian target of rapamycin 1 (mTORC1), a key downstream molecule of the PI3K/AKT pathway, accelerates IPF progression by promoting EMT and ECM protein synthesis [24]. The upstream regulators of mTORC1 are involved in excessive TGF‐β1‐induced proliferation of myofibroblasts and EMT, indicating that they can regulate the production of TGF‐β1, subsequently leading to the induction of EMT and proliferation of myoblasts [25]. Clinical trials of small‐molecule inhibitors targeting the PI3K/mTOR pathway for treating IPF patients have been conducted [26]. The PI3K/AKT pathway significantly affects the initiation and progression of IPF. Additionally, TRKA may significantly affect this pathway in IPF. Therefore, we speculated that TRKA may affect EMT and the proliferation of alveolar epithelial cells by controlling the PI3K/AKT pathway, thus influencing the progression of IPF.

In this study, we conducted RNA‐sequence analysis to investigate the regulatory molecules of YFT in BLM‐induced mice. We focused on the key molecule TRKA, which may be influenced by YFT. By examining the molecular mechanisms involved, we determined how YFT modulates the TRKA/PI3K/AKT pathway to affect the EMT and proliferation of alveolar epithelial cells.

## 2. Materials and Methods

### 2.1. IPF Mouse Model and YFT Treatment

C57BL/6 background mice (20 ± 2 g) were selected for the study, and they were randomly assigned to the sham group (*n* = 5); the remaining mice (*n* = 10) were used to establish IPF models. The mice were anesthetized with 0.6% sodium pentobarbital and subjected to intratracheal injection of BLM (5 mg/kg). These BLM‐induced mice were randomly divided into two groups: the BLM group (*n* = 5) received PBS, while the YFT group (*n* = 5) received YFT (11.2 g/kg/d) administered intragastrically. The dosage of YFT was adjusted based on values reported in previous studies [12]. After 21 days of intervention, the mice were euthanized with sodium pentobarbital (100 mg/kg ip), and their lungs were collected, fixed in 4% paraformaldehyde, and decalcified in 15% ethylenediamine tetraacetic acid (EDTA) before being prepared as paraffin‐embedded samples. The paraffin‐embedded blocks of lung tissue were processed into slices (4‐μm thick) for pathological analysis. The Animal Experimentation Ethics Review Committee of Yunnan University of Chinese Medicine approved this animal experiment with approval no. R‐062022G011, and Approval date: February 27, 2022.

### 2.2. Histopathological Analyses

Lung tissue pathology was examined using hematoxylin & eosin (H&E) and Masson staining methods. The lung slices were treated with Harris hematoxylin (G1004, Servicebio) for 8 min, eosin (G1002, Servicebio) dye for 3 min, and then sealed with neutral gum. For Masson staining, the lung slices were treated with Weigert’s iron hematoxylin and differentiated with acidic ethanol solution for a few seconds. Then, the slices were treated with a bluing solution for several seconds and stained with Ponceau dye (G2011, Servicebio). After treatment with phosphomolybdic acid solution, the tissues were restained with aniline blue dyeing solution (G1032, Servicebio). The slices were treated with an acetic acid working solution and then sealed after dehydration. A microscope was used to observe the stained samples.

### 2.3. Western Blotting Analysis

To extract total protein from lung tissues and cells, a lysate (P0013, Beyotime) was added to every 10 mg of clipped tissue. To extract total protein from the cells, the washed cells were treated with lysis buffer after the culture medium was removed. The extracted protein was subsequently quantified using the bicinchoninic acid method (Beyotime), and SDS‐PAGE was subsequently performed to separate the extracted proteins, which were subsequently transferred onto a nitrocellulose membrane. The membranes were pretreated with 5% nonfat milk and incubated with anti‐TGF‐β1 (AF1027, Affinity, 1:1000), antifibronectin (FN, 15613‐1‐AP, Proteintech, 1:10,000), anti‐alpha‐smooth muscle actin (α‐SMA, AF1032, Affinity, 1:500), anticollagen I (AF7001, Affinity, 1:500), anti‐E‐cadherin (20874‐1‐AP, Proteintech, 1:35,000), anti‐N‐cadherin (22018‐1‐AP, Proteintech, 1:10,000), anti‐PI3K (AF6241, Affinity, 1:1000), anti‐p‐PI3K (AF3242, Affinity, 1:1000), anti‐AKT (AF6261, Affinity, 1:1000), anti‐p‐AKT (AF0016, Affinity, 1:1000), anti‐TRKA (CY5331, Abways, 1:1000), anti‐p‐TRKA (AF3072, Affinity, 1:1000), and GAPDH (AB0036, Abways, 1:5000) antibodies overnight at 4°C. The samples were incubated with secondary antibodies. The protein expression was normalized to that of GAPDH.

### 2.4. RNA Sequencing

The lung tissue samples used for RNA sequencing were obtained from Beijing Novogene Co., Ltd. The lung tissue was mixed with TRIzol reagent to extract total RNA, and the integrity and quantity of RNA were determined. The quality of the cDNA library was assessed using an Agilent 2100 bioanalyzer after the synthesis of cDNA. The PCR products were subsequently sequenced on an Illumina platform, and the fluorescence signal of each cluster was collected and converted into the corresponding base, thus obtaining sequencing data.

### 2.5. Bioinformatics Analysis

The differentially expressed genes (DEGs) were selected with a set at *p*_adj_ < 0.05 and a |log2FoldChange| > 1. Gene Ontology (GO) enrichment and Kyoto Encyclopedia of Genes and Genomes (KEGG) pathway annotation analyses of these DEGs were performed using the clusterProfiler package in R. To better select the molecules regulated by YFT during the mitigation of lung fibrosis in BLM‐induced mice, the DEGs with stricter sets (*p*_adj_ < 0.05 and |log2FoldChange| > 2) were enriched.

### 2.6. Double Immunofluorescence Staining

Paraffin‐embedded slices were dewaxed with dewaxing solution (G1128, Servicebio) and rehydrated with ethyl alcohol. After antigen retrieval, endogenous peroxidase activity was blocked with 3% hydrogen peroxide. Serum blocking was performed with 3% bovine serum albumin (GC305010, Servicebio). Slices were incubated with antisurfactant protein C (SP‐C, 1:250, #DF6647, Affinity) overnight at 4°C. After washing with PBS, the slices were incubated with the secondary antibody, goat antimouse IgG HRP‐conjugate. Then, the slices were incubated with CY3‐tyramide (G1223, Servicebio), and antigen retrieval was performed. Slices were incubated overnight with anti‐TRKA (1:100, CY5331, Abways) at 4°C, followed by incubation with goat antimouse immunoglobulin‐G FITC‐conjugate. The slices were incubated with DAPI solution (G1012, Servicebio) and treated with an autofluorescence quencher (G1221, Servicebio). The slices were treated with antifade mounting medium (G1401, Servicebio).

### 2.7. Cell Culture

The human alveolar epithelial cell line A549 was purchased from Procell (CL‐0016). The cells were grown in RPMI‐1640 medium supplemented with 10% fetal bovine serum at 37°C with 5% CO_2_. Different concentrations of YFT (0, 100, 300, and 1000 μg/mL) were used to treat cells induced with TGF‐β1 (10 ng/mL, HY‐P70648, MCE) dissolved in DMSO. YFT (300 μg/mL) was used for the subsequent experiments. The collected culture supernatants were used to detect NGF concentrations with ELISA kits (F03104, YOYOBIO).

### 2.8. Quantitative Real‐Time PCR (RT‐qPCR)

The cells were initially mixed with TRIzol reagent (15,596,026, Invitrogen), followed by the addition of 200 μL of chloroform for phase separation, and isopropanol was added to precipitate RNA from the aqueous phase. A reverse transcription kit (R333‐01, Vazyme) was used to perform reverse transcription. Then, qPCR was subsequently performed via ChamQ SYBR Mix (Q711‐02, Vazyme). The primers used were as follows: TRKA‐F: 5′‐ATC​TTG​ACG​GTG​AAG​TCC​T‐3′; TRKA‐R: 5′‐GTT​CTC​GAT​GTA​GCT​TGC​TG‐3′; GAPDH‐R: 5′‐ CAT​CCA​CAG​TCT​TCT​GGG​TG‐3′; and GAPDH‐F: 5′‐GTT​CGT​CAT​GGG​TGT​GAA​CC‐3′. GAPDH was used as an internal reference for mRNA quantification, and the relative level of TRKA expression was computed by the 2^–ΔΔCt^ method.

### 2.9. EdU Assay

EdU assays were performed using a BeyoClick EdU‐488 cell proliferation kit (C0071S) following the manufacturer’s instructions. A549 cells were treated with the prepared dEdU solution at 37°C for 2 h. After labeling, the cells were fixed, treated with 0.3% Triton X‐100, and subsequently treated with a click additive solution. Then, Hoechst 33,342 was added, and a microscope was used to observe the results.

### 2.10. Cell Transfection

To overexpress TRKA, TRKA was synthesized by BGI and cloned and inserted into the pcDNA3.1 (+) (Invitrogen, USA) vector, which contains EcoRI and HindIII restriction sites. The recombinant plasmid or pcDNA3.1(+) vector was subsequently amplified and verified by agarose gel electrophoresis. A549 cells were cultured in a 24‐well plate and grown to 70%–80% confluence. A549 cells were transfected with the TRKA recombinant plasmid or the pcDNA3.1 (+) vector with Lipofectamine 2000 (11,668,019, Thermo Scientific). The transfection mixture was removed after 6 h of transfection, and the cells were cultured with complete medium and subjected to different treatments.

### 2.11. Statistical Analysis

All statistical analyses were conducted using GraphPad Prism 8.0 (GraphPad Software, USA), and the results are presented as the mean ± standard deviation (SD). The differences between groups were analyzed, and Student’s *t*‐tests between two groups and one‐way ANOVA followed by Tukey’s post hoc test among groups were conducted. All differences were considered to be statistically significant at *p* < 0.05.

## 3. Results

### 3.1. YFT Intervention Mitigated Lung Fibrosis in BLM‐Induced IPF Mice

The H&E‐stained and Masson tricolor‐stained sections revealed that, compared to those of the sham group, the lung structure of the BLM group was damaged, characterized by excessive collagen deposition and fiber hyperplasia (Figure 1(a)). YFT significantly reduced the pathological injuries observed in the lung tissue. In the lung tissue, BLM considerably increased the levels of TGF‐β1, FN, α‐SMA, and collagen I, which were substantially reduced after YFT treatment (Figure 1(b)). We conducted a transcriptomic analysis of lung tissues from BLM‐induced mice treated with YFT. The DEGs were identified using cutoff values of *p*_adj_ < 0.05 and |log2fold change| > 1. Compared to those in the sham group, 1627 downregulated and 1565 upregulated DEGs were identified in the model group. Compared to those in the BLM group, 847 downregulated and 1712 upregulated DEGs were identified in the YFT group (Figure S1).

Figure 1YFT attenuated lung fibrosis in a BLM‐induced mouse model. (a) Representative images of hematoxylin and eosin staining and Masson’s trichrome staining of left lung slices. (b) Western blotting analysis was conducted to examine fibrotic markers, including TGF‐β1, fibronectin (FN), α‐SMA, and collagen I.  ^∗∗^ *p* <  0.01, compared to the sham group; ^#^ *p* <  0.05, compared to the BLM group; ^##^ *p* <  0.01, compared to the BLM group.

**Figure:**
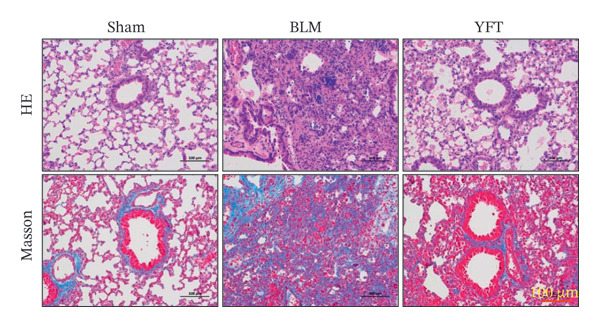
(a)

**Figure:**
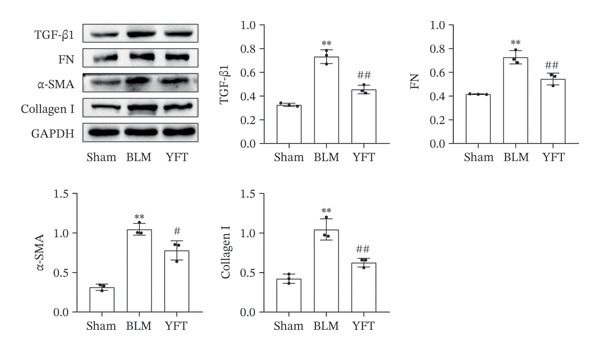
(b)

### 3.2. Transcriptomic Analysis of Lung Tissues From BLM‐Induced Mice Treated With YFT

We conducted a transcriptomic analysis on lung tissues obtained from the model animals in each group. The DEGs were identified using cutoff values of *p*_adj_ < 0.05 and |log2fold change| > 1. Compared to those in the sham group, 1627 downregulated and 1565 upregulated DEGs were identified in the model group. Compared to those in the BLM group, 847 downregulated and 1712 upregulated DEGs were identified in the YFT group (Figure S1).

According to the GO annotations of these DEGs from the BLM group versus the sham group (Figure 2(a)), the DEGs were involved primarily in biological processes related to angiogenesis and positive regulation of cell migration. Most of the annotations for cellular components were concentrated in the ECM and proteinaceous ECM. With respect to molecular functions, receptor regulator activity and receptor ligand activity were the main enriched terms. The biological processes of the DEGs identified between the YFT and BLM groups were enriched primarily in the regulation of leukocyte activation and T‐cell activation. Annotations related to cellular components were observed mainly in the ECM and proteinaceous ECM. The molecular function annotations were enriched mainly in sulfur compound binding and peptidase regulator activity. To identify the pathways associated with the DEGs, a KEGG pathway analysis was performed. The DEGs identified between the BLM and sham groups were enriched in pathways such as the PI3K–AKT signaling pathway, cytokine–cytokine receptor interaction, cell adhesion molecules (CAMs), the Rap1 signaling pathway, and focal adhesion (Figure 2(b)). For the DEGs identified between the YFT and BLM groups, the annotations were most associated with the PI3K–AKT signaling pathway, the Ras signaling pathway, focal adhesion, cytokine–cytokine receptor interaction, and CAMs.

Figure 2GO and KEGG pathway enrichment analyses were performed on DEGs in two comparisons: the BLM group versus the sham group and the YFT group versus the BLM group. (a) GO analysis was conducted using the biological process, cellular component, and molecular function categories for both comparisons. (b) KEGG pathway enrichment analysis was performed on the DEGs for both comparisons.

**Figure:**
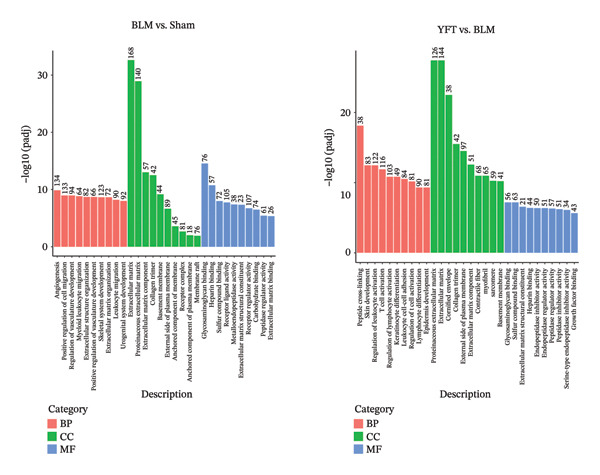
(a)

**Figure:**
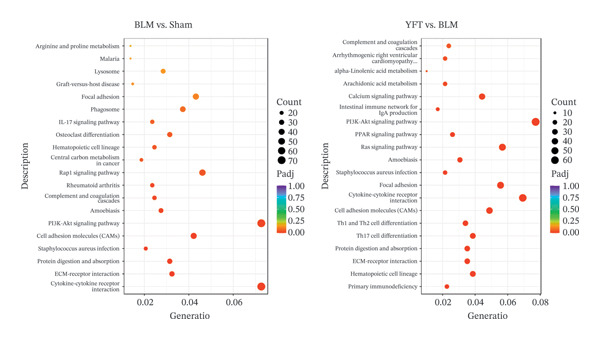
(b)

### 3.3. YFT Reduced the Number of SP‐C^+^/TRKA^+^ Cells in BLM‐Induced Lung Tissues and Inhibited TRKA Expression in TGF‐β1‐Induced Alveolar Epithelial Cells

To select the molecules regulated by YFT mitigating lung fibrosis in BLM‐induced mice, we examined the DEGs shared by the BLM group versus the PBS group and the YFT group versus the BLM group, focusing on |log2foldchange| > 2 DEGs (Table 1). Among these DEGs, the expression of TRKA increased after BLM induction and YFT treatment. Consequently, we decided to use TRKA as a molecule for further investigation to determine whether YFT affects alveolar epithelial cells by regulating TRKA. First, we conducted double immunofluorescence staining to test the co‐localization of TRKA with alveolar epithelial cells (SP‐C^+^) (Figure 3(a)). The results revealed that TRKA co‐localized with SP‐C and that YFT reduced the number of SP‐C^+^/TRKA^+^ cells in BLM‐induced mice. In vitro cell experiments, we stimulated A549 cells with TGF‐β1, which increased TRKA mRNA and protein levels (Figures 3(b) and 3(c)). However, YFT reduced the levels of TRKA mRNA and protein in A549 cells in a dose‐dependent manner. Additionally, TGF‐β1 increased the concentration of the TRKA ligand NGF in A549 cells, whereas YFT decreased the concentration of NGF in a dose‐dependent manner (Figure 3(d)).

**Table 1:** The shared differentially expressed genes (DEGs) between BLM versus PBS and YFT versus BLM.

	**BLM vs. PBS**		**YFT vs. BLM**	
	**log2FC**	**Adjusted** **p** **-value**	**log2FC**	**Adjusted** **p** **-value**

*Gpt*	−2.52376	1.96E − 14	2.112014	4.41E − 05
*Adra2b*	2.197591	0.003828	−3.23097	0.000211
*Mmp8*	2.94884	2.23E − 17	−2.02083	3.29E − 05
*F13a1*	4.290146	1.11E − 29	−2.41786	4.19E − 07
*Trpv3*	−2.81027	0.013911	4.065554	0.000369
*TRKA*	2.009733	0.045614	2.061451	0.03158

Figure 3YFT reduced the number of SP‐C^+^/TRKA^+^ cells in BLM‐induced lung tissues and inhibited the expression of TRKA in TGF‐β1‐induced alveolar epithelial cells. (a) Double immunofluorescence staining of TRKA and SP‐C in lung slices from BLM‐induced model mice. (b) TRKA mRNA expression was detected by RT‐qPCR in A549 cells after TGF‐β1 stimulation. (c) Western blotting analysis of TRKA in A549 cells was conducted after TGF‐β1 stimulation. (d) ELISA was conducted to detect the concentration of NGF.  ^∗∗^ *p* <  0.01, compared to DMSO; ^##^ *p* <  0.01, compared to TGF‐β1.

**Figure:**
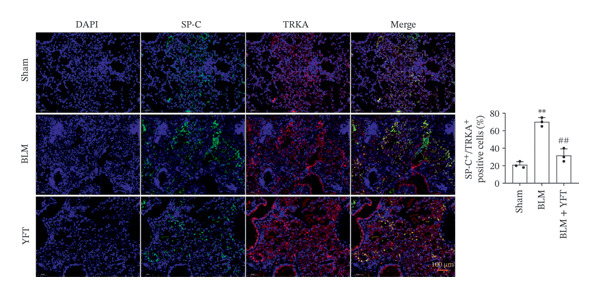
(a)

**Figure:**
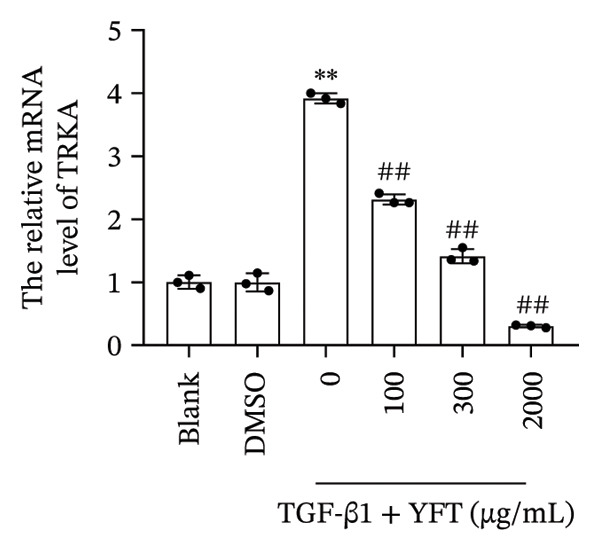
(b)

**Figure:**
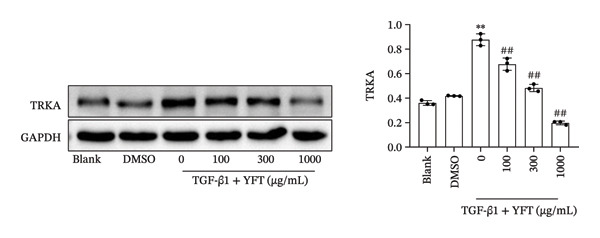
(c)

**Figure:**
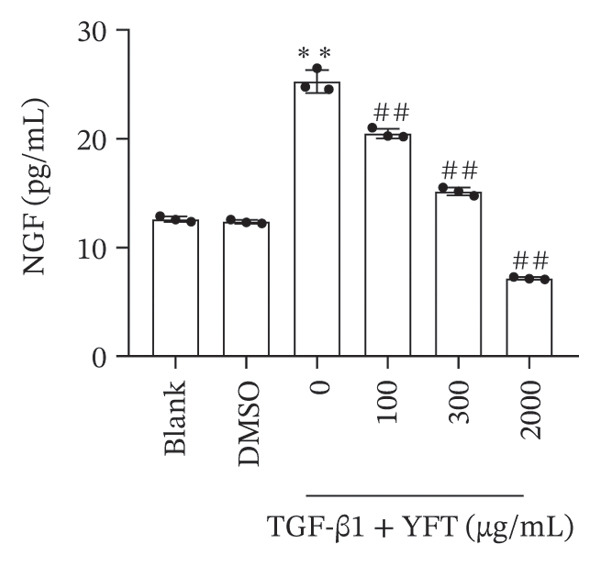
(d)

### 3.4. YFT Inhibited the EMT and Proliferation of TGF‐β1‐Induced Alveolar Epithelial Cells

We further investigated the effect of YFT (300 μg/mL) on the EMT and proliferation of A549 cells. TGF‐β1 stimulation decreased E‐cadherin protein levels in A549 cells. Moreover, it increased the protein levels of N‐cadherin, α‐SMA, FN, and collagen I in A549 cells (Figure 4(a)). In contrast, YFT treatment resulted in an increase in E‐cadherin protein levels and a decrease in N‐cadherin, α‐SMA, FN, and collagen I protein levels. Moreover, the presence of TGF‐β1 increased the number of EdU + A549 cells, whereas the effect of TGF‐β1 decreased in the presence of YFT (Figure 4(b)).

Figure 4YFT suppressed the EMT and proliferation induced by TGF‐β1 in A549 cells. (a) Western blotting analysis was conducted to examine EMT marker proteins (E‐cadherin, N‐cadherin, and α‐SMA) and ECM markers (FN and collagen I) in A549 cells after TGF‐β1 stimulation. (b) YFT decreased the proliferation of A549 cells after TGF‐β1 stimulation.  ^∗^ *p* <  0.05, compared to DMSO;  ^∗∗^ *p* <  0.01, compared to DMSO; ^#^ *p* <  0.05, compared to TGF‐β1 + YFT (0 μg/mL); ^##^ *p* <  0.01, compared to TGF‐β1 + YFT (0 μg/mL).

**Figure:**
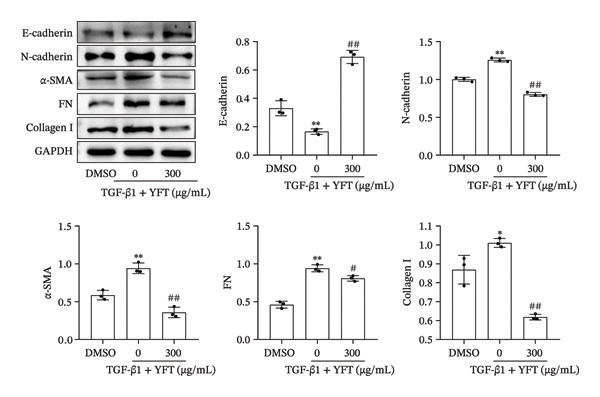
(a)

**Figure:**
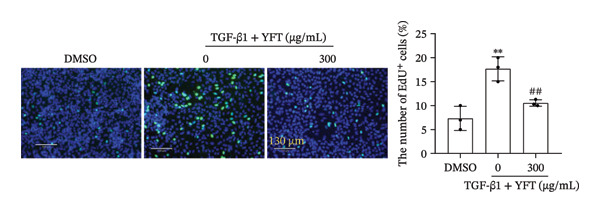
(b)

### 3.5. YFT Inhibited the TRKA/PI3K/AKT Pathway to Restrain the EMT and Proliferation of TGF‐β1‐Induced Alveolar Epithelial Cells

To investigate the effect of YFT on the PI3K/AKT pathway and the role of TRKA in this process, we constructed A549 cells overexpressing TRKA (Figure 5(a)). The overexpression of TRKA promoted NGF expression, and YFT reduced the level of TGF‐β1‐induced NGF, whereas the overexpression of TRKA reduced the effect of YFT (Figure 5(b)). Similarly, TRKA overexpression promoted p‐TRKA, TRKA, p‐PI3K, and p‐AKT levels, and YFT reduced the TGF‐β1‐induced levels of p‐TRKA, TRKA, p‐PI3K, and p‐AKT in A549 cells, whereas the overexpression of TRKA increased the levels of these molecules (Figures 5(c) and 5(d)). These findings suggested that YFT regulates the activation of the PI3K/AKT signaling pathway through TRKA.

Figure 5YFT suppressed TRKA to inhibit the PI3K/AKT signaling pathway in A549 cells induced with TGF‐β1. (a) RT‐qPCR was performed to detect the mRNA level of TRKA. (b) ELISA was conducted to detect the concentration of NGF. (c and d) Western blotting analysis was performed to examine TRKA, p‐TRKA, p‐PI3K, PI3K, p‐AKT, and AKT levels in A549 cells.  ^∗∗^ *p* <  0.01, compared to the Vector; ^##^ *p* <  0.01, compared to the Control; ^$$^ *p* <  0.01, compared to the Vector + YFT.

**Figure:**
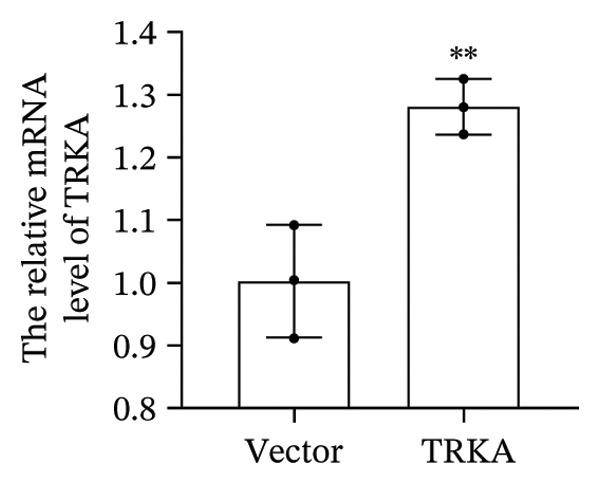
(a)

**Figure:**
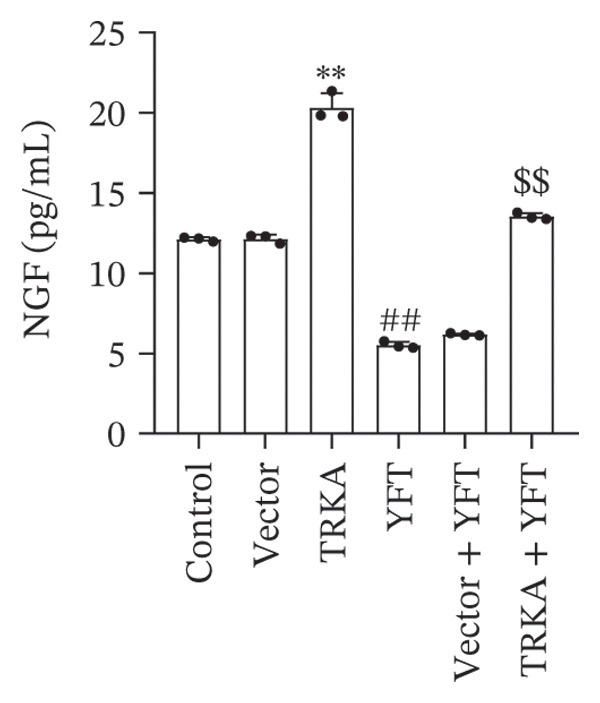
(b)

**Figure:**
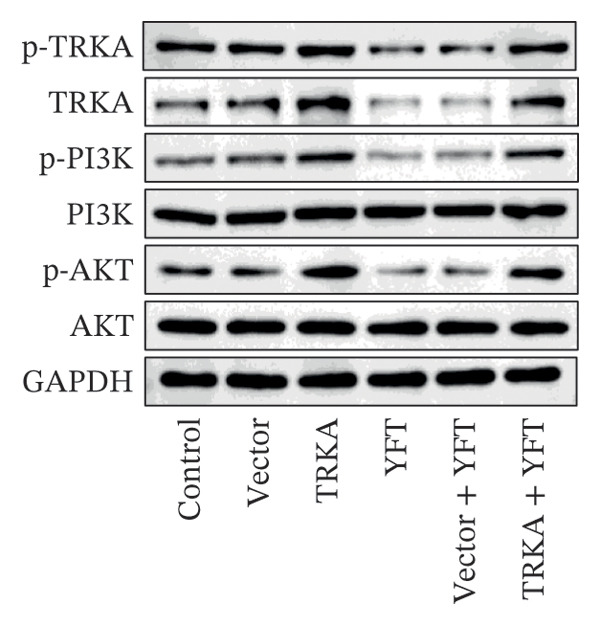
(c)

**Figure:**
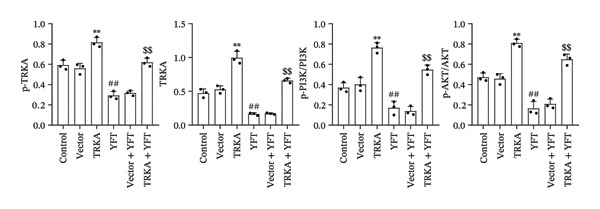
(d)

Upregulated TRKA resulted in a reduction in E‐cadherin expression and an increase in N‐cadherin, α‐SMA, FN, and collagen I protein expression (Figure 6(a)). In contrast, YFT increased E‐cadherin protein expression and decreased N‐cadherin, α‐SMA, FN, and collagen I protein expression in TGF‐β1‐induced A549 cells. However, the overexpression of TRKA decreased the level of E‐cadherin protein and increased the levels of N‐cadherin, α‐SMA, FN, and collagen I proteins. We also constructed TRKA‐knockdown A549 cells (Figures S2A and S2B). Knocking down TRKA increased the level of E‐cadherin protein and decreased the levels of N‐cadherin, α‐SMA, FN, and collagen I proteins in TGF‐β1‐induced A549 cells (Figures S2C). Similarly, treatment with YFT also suppressed EMT in TGF‐β1‐induced A549 cells.

Figure 6YFT suppressed TRKA to inhibit the EMT and proliferation of A549 cells induced by TGF‐β1. (a) Western blotting analysis was performed to determine the levels of E‐cadherin, N‐cadherin, α‐SMA, FN, and collagen I in A549 cells. (b) The proliferation of A549 cells was measured by an EdU proliferation assay.  ^∗∗^ *p* <  0.01, compared to the Vector; ^##^ *p* <  0.01, compared to the Control; ^$$^ *p* <  0.01, compared to the Vector + YFT.

**Figure:**
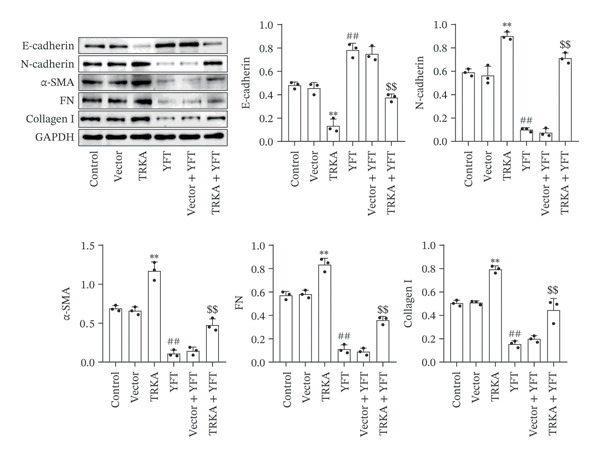
(a)

**Figure:**
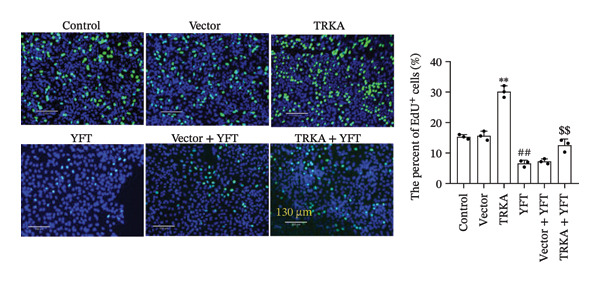
(b)

Additionally, TRKA overexpression promoted the number of EdU^+^ A549 cells induced by TGF‐β1, and YFT decreased the number of EdU^+^ A549 cells induced by TGF‐β1, whereas TRKA overexpression reduced the effect of YFT (Figure 6(b)). These results showed that YFT might restrain the EMT and proliferation of TGF‐β1‐induced A549 cells by inhibiting the TRKA/PI3K/AKT pathway.

## 4. Discussion

In this study, we investigated the therapeutic effect of YFT on IPF and investigated its molecular mechanism of action. Our findings demonstrated that YFT effectively reduced BLM‐induced lung fibrosis in mice. Previous studies have shown that nidanib and pirfenidone (antifibrotic drugs) are safe and effective for treating IPF [27–29]. In our study, YFT decreased the levels of ECM proteins (fibronectin and collagen I), α‐SMA, and TGF‐β1 in lung tissues. The stiffening of lung tissue caused by the deposition of ECM is a significant feature of pulmonary fibrosis, leading to impaired lung expansion and oxygen exchange [30, 31]. The ECM, which is composed of collagen, elastin, proteoglycans, and noncollagen glycoproteins, provides structural support for cells and mechanical support for lung function [32]. Fibronectin is abnormally abundant in regions where fibrosis occurs [33]. Additionally, compared to that in normal lung tissue, collagen I expression is higher in fibrotic lung tissue, contributing to greater tissue hardness [34]. TGF‐β overexpression can induce pulmonary fibrosis in mice with mild inflammation [35]. TGF‐β binds to its receptor and promotes the phosphorylation of the transcription factor Smad3, resulting in the expression of target genes such as fibronectin, collagen I, and α‐SMA [36–38]. Therefore, YFT may be a promising treatment option for pulmonary fibrosis, functioning as a potential antifibrotic medication.

To determine whether a drug acts in an inflammatory or fibrotic phase, the time of administration needs to be considered. However, the inflammatory and fibrotic phases in the BLM model are overlapping, with no clear distinction between the two phases [39]. Therefore, separating the inflammatory and fibrotic phases by the accepted 7 days has certain limitations [39]. Early treatment not only reduces damage and inflammation in the model but also influences the development of fibrosis in the model. Therefore, in our study, an IPF model was constructed using BLM and administered one day after induction to evaluate the efficacy and mechanism of YFT in treating IPF. As the mechanism of action of YFT was initially unclear, YFT administration was started early, with transcriptome sequencing after 28 days to identify regulated genes. Based on the animal experimental results obtained thus far, it remains uncertain whether YFT can directly inhibit the initial inflammatory response during early treatment. This study revealed that YFT can inhibit EMT induced by TGF‐β1 in epithelial cells through in vitro experiments, suggesting that YFT may be effective in treating IPF. However, further experiments are needed to investigate the effects of YFT in both the inflammatory and fibrosis stages. Future studies should focus on designing different administration times to determine the optimal stage for YFT treatment in IPF. Additionally, animal experiments are necessary to investigate whether YFT has preventive, therapeutic, or combined effects on IPF.

To further study the molecular mechanism by which YFT alleviates IPF, we conducted transcriptome sequencing on the lung tissues of mice induced with BLM and treated with YFT. We specifically focused on TRKA, a molecule that YFT may affect. According to the transcriptome sequencing results of the tissue samples, the expression of TRKA increased in the BLM group and further increased in the BLM + YFT group. This finding was inconsistent with the results of the in vitro experiments. However, the data and conclusions concerning the expression of TRKA in epithelial cells from lung tissues and A549 epithelial cells were consistent. Additionally, TRKA expression was also observed in other cells in the tissue. Further research is needed to identify which cells express TRKA and how TRKA was regulated by YFT. Additionally, YFT decreased the levels of NGF, TRKA, and p‐TRKA in the TGF‐β1‐induced A549 cells. These findings imply that YFT might inhibit the activation of NGF/TRKA in lung epithelial cells, thereby treating IPF. When kidney cells are exposed to a hyperglycemic environment, TRKA is phosphorylated in mesangial cells, tubular epithelial cells, and podocytes. However, this phosphorylation does not occur in glomerular endothelial cells or kidney fibroblasts [18]. Therefore, the activation of TRKA may vary depending on the specific cellular environment.

Studies have found that NGF/TRKA can activate multiple pathways. When NGF binds to TRKA, it induces autophosphorylation of the kinase domain, creating binding sites for various scaffold proteins that facilitate the activation of intracellular signaling pathways (MAPK and PI3K signaling cascades) [40]. For example, TRKA can activate the p38/ERK MAPK signaling pathway to promote the proliferation and inflammation of rat mesangial cells [41]. Moreover, the PI3K/MAPK signaling pathway can be activated by the interaction of NGF and TRKA, which leads to the inhibition of apoptosis, stimulation of neuronal differentiation and axon growth, and facilitation of allergic reactions [42]. In human lens epithelial cells exposed to high concentrations of dexamethasone, NGF/TRKA can also target and activate the AKT pathway [43]. Additionally, the protective effect of NGF on apoptotic stimulation in NSC is attributed to the TRK receptor‐mediated PI3K/AKT signaling pathway [44]. We found that YFT inhibited the activation of the PI3K/AKT pathway in TGF‐β1‐induced A549 cells. TRKA overexpression reversed the inhibitory effect of YFT, indicating that YFT inhibits the activation of the PI3K/AKT pathway by targeting NGF/TRKA. However, TRKA does not significantly affect the activation of the AKT signaling pathway in mesangial cells [41]. These findings suggest that NGF/TRKA‐mediated activation of the PI3K/AKT pathway may depend on the type of cell.

In addition, in this study, DEGs from both the BLM versus sham and the YFT versus BLM comparisons were enriched in CAMs. The expression of these molecules was a hallmark of the EMT. Many studies have reported that TGF‐β1 can induce both EMT and activation of the proliferation of A549 cells [45–47]. PI3K–mTOR signaling is consistently activated in TGF‐β1‐induced A549 cells, as shown by elevated levels of phosphorylated mTOR and phosphorylated S6, which promote cell proliferation [45]. In this study, TGF‐β1 also induced the proliferation of A549 cells, and YFT inhibited the activation of the NGF/TRKA/PI3K/AKT pathway to regulate cell proliferation. However, treatment of A549 cells with TGF‐β can inhibit cell proliferation and cell cycle progression [48]. This discrepancy in results indicates the need for further experiments to validate the findings.

A study showed that calpain 1 regulated EMT in TGF‐β1‐induced A549 epithelial cells through the PI3K/AKT signaling pathway [49]. Another study revealed that TGF‐β1 stimulates the activation of the PI3K/AKT pathway in A549 cells, resulting in hyperphosphorylation and downregulation of forkhead box transcription factor 3a (FOXO3a), ultimately inducing EMT [50]. Similarly, glycyrrhizin can inhibit EMT in A549 cells by inhibiting high mobility group protein 1 (HMGB1)/brahma‐related gene 1 (BRG1) expression through the PI3K/AKT/mTOR pathway [51]. Therefore, TGF‐β1 may induce the EMT and proliferation of A549 cells by the activation of the PI3K/AKT/mTOR pathway. Our results revealed that YFT inhibited the activation of the NGF/TRKA/PI3K/AKT pathway in TGF‐β1‐induced A549 cells, leading to the inhibition of EMT. These findings indicate that YFT may have potential as an inhibitor that targets the PI3K pathway in the treatment of IPF. NGF interacts with TRKA and p75 (NTR) in HK‐2 renal tubule cells to regulate the expression and secretion of TGF‐β1 and increase the levels of EMT markers, consequently contributing to renal fibrosis [52]. The NGF/TRKA axis triggers EMT through STT3 activation, thereby promoting the migration of HNSCC cells [53]. However, a study revealed that the activation of NGF/TRKA can effectively hinder EMT in human lens epithelial cells when they are subjected to high concentrations of dexamethasone [43]. Therefore, the regulation of PI3K/AKT/EMT by NGF/TRKA may vary across pathological conditions and cells. Moreover, a clinical study involving IPF patients tested the safety and tolerability of omipalisib, a PI3K/mTOR inhibitor, which has promising pharmacodynamic activity and acceptable patient tolerance [26]. These findings further support the potential use of YFT as a therapeutic agent for inhibiting the PI3K signaling pathway in lung epithelial cells during IPF treatment.

Small‐molecule inhibitors targeting TRK can be categorized into three types based on their binding sites as follows: Type I, Type II, and Type III. Type I inhibitors, such as larotrectinib, belizatinib, and K252a, are molecules that compete with ATP [54–56]. Type II TRK inhibitors, such as altiratinib and cabozantinib, are safer and more selective against TRK due to their nonconserved allosteric sites [57, 58]. VMD‐928 is a Type III TRK inhibitor, which is a biallosteric and irreversible selective inhibitor of TRKA, exhibiting minimal to no activity against 348 other kinases, such as TRKB and TRKC [59]. However, the inhibition of TRKA enzyme activity by this inhibitor affects all downstream pathways of TRKA. Therefore, a more effective approach to reduce side effects may involve specifically preventing TRKA from binding to a downstream pathway substrate. A study used a silicon model to identify key mutations affecting the interaction between TRKA and different substrates and synthesized a peptide based on the TRKA anchoring region to phospholipase Cγ (PLCγ), which inhibits the binding of TRKA to PLCγ and decreases mechanical sensitivity in models of inflammatory acute and chronic pain [60]. In this study, YFT not only reduced TRKA expression but also decreased the phosphorylation of p‐TRKA. Therefore, YFT may inhibit the transcription of TRKA or act as an inhibitor of NGF/TRKA activation, resulting in the inhibition of the downstream activation of the PI3K/Akt pathway. However, further studies are needed to determine whether the activation of other pathways is also inhibited and elucidate the underlying mechanism of inhibition.

Transcriptome sequencing identified other molecules that YFT might target. These molecules include glutamic–pyruvic transaminase (GPT), matrix metalloproteinase 8 (MMP8), coagulation factor XIIIA (F13A1), and transient receptor potential vanilloid subtype 3 (TRPV3). The downregulation of GPT intensifies the accumulation of abnormal collagen in vascular smooth muscle cells and promotes fibrosis [61]. In vitro experiments have also demonstrated that increased expression of TRPV3 induced by treatment with the TRPV3 agonists carvacrol or angiotensin II can promote the proliferation of neonatal cardiac fibroblasts and the expression of TGF‐β1 and collagen I [62]. Additionally, a study found that F13a1 knockout mice develop cardiac fibrosis [63]. Therefore, YFT may also target these molecules, thereby regulating pulmonary fibrosis in IPF. Future studies need to investigate the molecular mechanisms through which YFT affects these molecules.

IPF is a chronic disease characterized by a dysregulated response to alveolar epithelial injury, and abnormalities in the injury and repair processes of alveolar epithelial cells play a key role in the occurrence and development of IPF [1, 64]. Based on these facts, in this study, we focused on investigating alveolar epithelial cells in the context of IPF. Although immortalized cell lines derived from normal tissue, such as the bronchial epithelial cell line 16HBE, are commercially available, we decided not to use these bronchial epithelial cells for our research. A549 cells are frequently used as an in vitro model to study lung epithelial cell injury and the progression of EMT [65]. They are interesting because they can induce direct and indirect transformation of alveolar epithelial cells into fibroblasts or myofibroblasts [65]. When stimulated by TGF‐β1 and other factors, A549 cells can undergo stable EMT, making them highly suitable for investigating EMT mechanisms and assessing potential intervention strategies [66, 67]. Additionally, A549 cells are easy to culture and manipulate in vitro; they can adapt well to various experimental procedures such as transfection, drug treatment, and gene knockout [68]. The high availability and good batch‐to‐batch consistency of these methods contribute to the reproducibility of related experiments. Human‐derived primary alveolar epithelial cells (hPAEpCs) have become commercially available and are used in research on lung cell culture. However, many of their characteristics remain largely unknown [69, 70]. Many studies have investigated the morphology, surface marker expression, transcriptomic profiles, and functional properties of hPAEpCs [71]. These findings indicate that early‐passaged hPAEpCs most closely resemble the in situ alveolar epithelial phenotype. However, these cells quickly become unsuitable for use, as they are already at passage three (P3) when purchased. Consequently, direct comparison with freshly isolated counterparts is not feasible, and analyses in the present study were restricted to cells at passage five (P5) or higher. As the number of passages increases, these cells rapidly transition into alveolar‐basal intermediates, which maintain barrier function but lack typical markers of ATII cells. Therefore, hPAEpCs can generally be expanded for only three to five passages. The isolation and cultivation of primary cells is also relatively expensive and shares common drawbacks with other primary cell types, such as strict environmental requirements and demanding experimental procedures [72]. In the future, we may isolate or purchase normal epithelial cells to further validate our conclusions.

This study had several limitations. The animal sample size was small and needs to be increased further to confirm the curative effect of YFT on the pulmonary fibrosis of BLM‐induced mice. Additionally, the active components of YFT that reach the lungs differ from those in the decoction. Metabolite derivatives of YFT components may also significantly affect the overall effectiveness of the treatment. Owing to limited time and money, we did not design relevant experiments. However, some active ingredients in Chinese medicine can be directly absorbed into the bloodstream without the need for metabolism. Our experimental results showed good agreement between the in vitro and in vivo results, indicating the effectiveness of YFT. This partially demonstrates the role of YFT. In the future, we need to conduct many experiments to study the YFT components and metabolite derivatives, identify the substances that play a role, and understand the related molecular mechanisms. Although we demonstrated that YFT reduces the expression and phosphorylation levels of TRKA, how YFT achieves this goal is still unclear and requires further investigation. Myofibroblasts and fibroblasts are also important in the occurrence and development of IPF. NGF triggers the apoptosis of conjunctival myofibroblasts in vitro, mainly via p75NTR [73]. In the future, we may further investigate whether YFT also influences the cellular behavior of myofibroblasts and fibroblasts by acting on TRKA. Since many inhibitors of TRKA and PI3K signaling pathways have been discovered, the effectiveness and safety of combining YFT with these inhibitors need to be determined.

## 5. Conclusion

To summarize, the results of this study suggest that YFT regulates the NGF/TRKA/PI3K/AKT signaling pathway, thus reducing the EMT and proliferation of pulmonary epithelial cells. Our research offers a new perspective on the therapeutic mechanism of YFT in IPF, which can lead to a more comprehensive understanding of the pathogenesis of the disease.

**Nomenclature**
IPF: Idiopathic pulmonary fibrosis
ECM: Extracellular matrix
EMT: Epithelial–mesenchymal transformation
YFT: Yifei decoction
DEGs: Differentially expressed genes
NGF: Nerve growth factor
MAPK: Mitogen‐activated protein kinase
mTORC1: Mammalian target of rapamycin 1
H&E: Hematoxylin & eosin
BCA: Bicinchoninic acid
PLCγ: Phospholipase Cγ.

## Author Contributions

All the authors were involved in this study. Lijuan Chen analyzed and interpreted the data and was a major contributor in writing the manuscript. Chengzhong Lan conceived and designed the study and was a major contributor in critically revising the manuscript. Xiangli Deng, Mei Wu, Mei Shao, Mei Yang, and Xinwen Ma were responsible for data analysis and visualization and literature search. Fen Huang and Haiyin Wu were responsible for data curation and visualization.

## Funding

This study was supported by the Regional Science Fund Program of National Natural Science Foundation of China (Grant no.: 82360922), Joint Special Project for Applied Basic Research for the Yunnan Provincial Science and Technology Department‐Yunnan University of Traditional Chinese Medicine (Youth Project) [Grant no.: 2018FF001(‐080)], and Joint Special Project for Applied Basic Research for the Yunnan Provincial Science and Technology Department‐Yunnan University of Traditional Chinese Medicine (General Program) [Grant no.: 2019FF002(‐034)].

## Disclosure

All authors have read and agreed to the final version and are accountable for all aspects of the work.

## Ethics Statement

This study was approved by the Animal Experimentation Ethics Review Committee of Yunnan University of Chinese Medicine (approval no.: R‐062022G011, approval date: February 27, 2022) in accordance with the guidelines and regulations for laboratory animals.

## Conflicts of Interest

The authors declare no conflicts of interest.

## Supporting Information

Additional supporting information can be found online in the Supporting Information section.

## Supporting information


**Supporting Information 1** Figure S1: The number of DEGs between the BLM and PBS groups and between the BLM and YFT groups was determined. The upregulated and downregulated DEGs were defined based on *p*_adj_ (< 0.05) and |log2fold change| > 1.

**Supporting Information 2** Figure S2: TRKA knockdown reduced the EMT of A549 cells induced with TGF‐β1. A. RT‐qPCR was performed to detect the mRNA level of TRKA. B. Western blotting analysis was used to detect the protein level of TRKA. C. Western blotting analysis was performed to determine the levels of E‐cadherin, N‐cadherin, α‐SMA, FN, and collagen I.  ^∗∗^ *p* <  0.01, compared to the Si‐NC; ^##^ *p* <  0.01, compared to the Si‐TRKA.

## Data Availability

The data that support the findings of this study are available from the corresponding author upon reasonable request.
