# Supplementary figures and images for: Yifei Decoction Regulates the NGF/TRKA/PI3K/AKT Signaling Axis to Inhibit the Epithelial–Mesenchymal Transformation and Proliferation of Pulmonary Epithelial Cells in Bleomycin‐Induced Pulmonary Fibrogenesis
Source: Can Respir J. 2026 Jan 30;2026:6614209. doi: 10.1155/carj/6614209 (PMC12856217; doi:10.1155/carj/6614209)

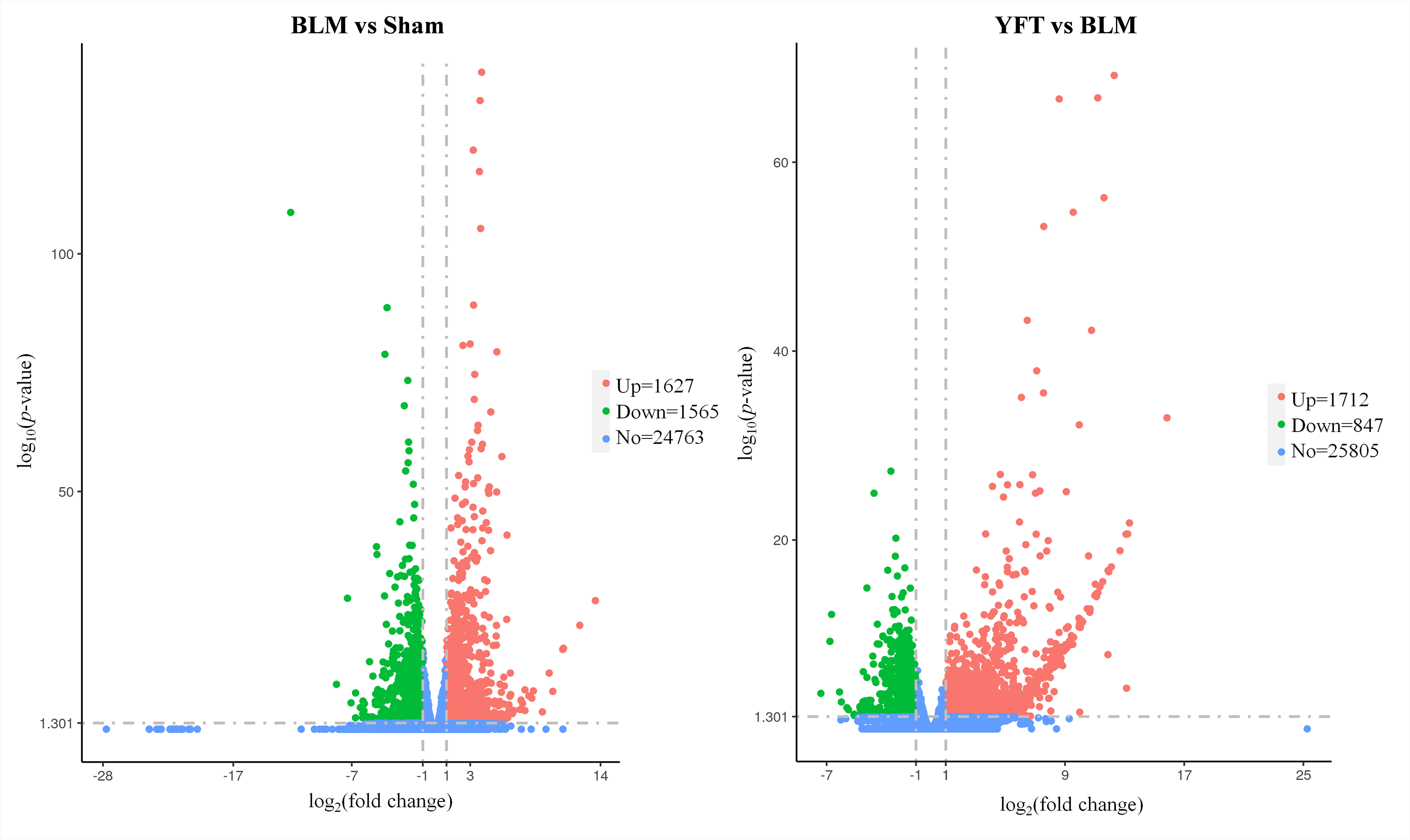

Supplement: Supplementary file 1 — Supporting Information 1 Figure S1: The number of DEGs between the BLM and PBS groups and between the BLM and YFT groups was determined. The upregulated and downregulated DEGs were defined based on p adj (< 0.05) and |log2fold change| > 1. [file CARJ-2026-6614209-s002.tif]

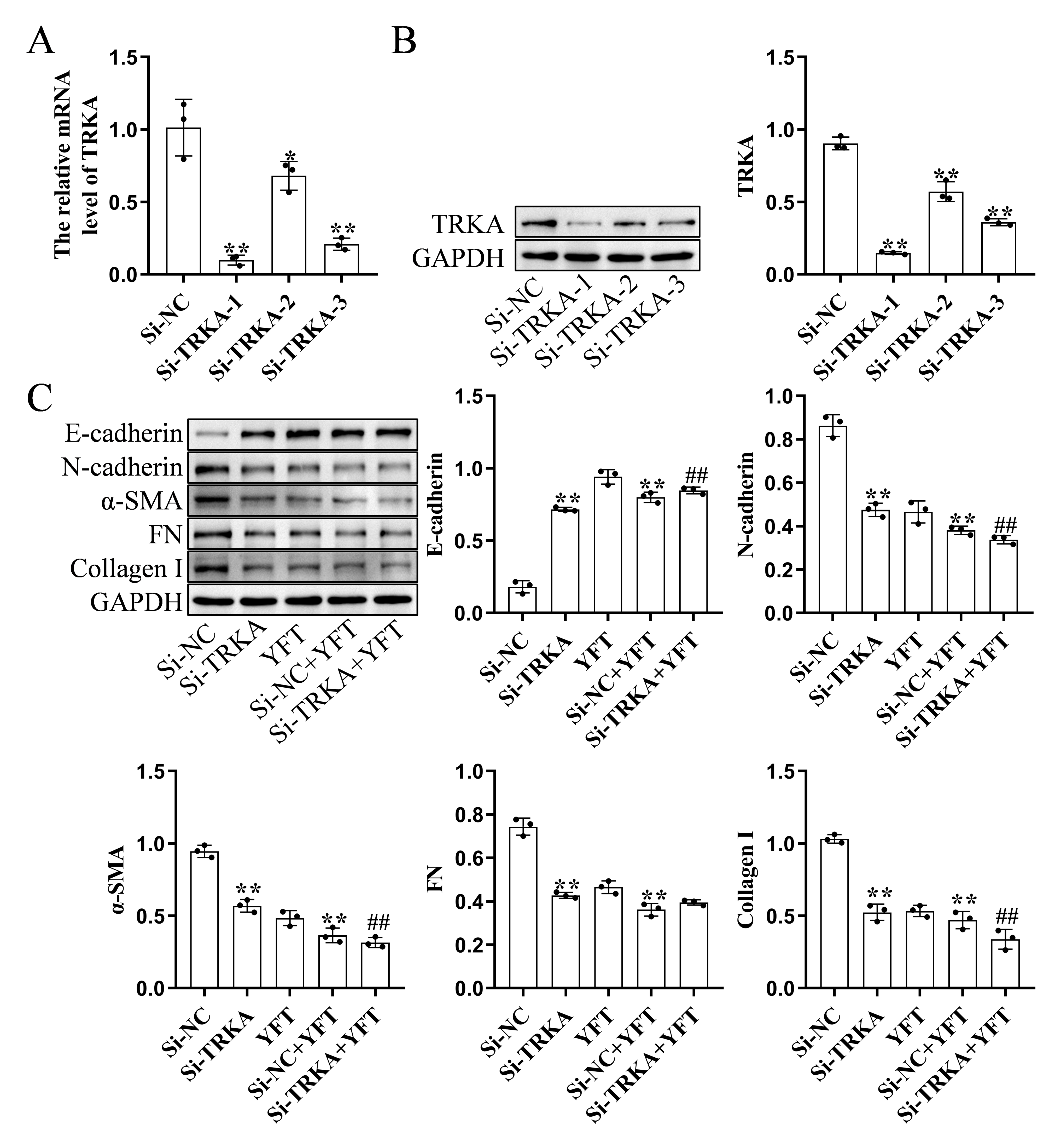

Supplement: Supplementary file 2 — Supporting Information 2 Figure S2: TRKA knockdown reduced the EMT of A549 cells induced with TGF‐β1. A. RT‐qPCR was performed to detect the mRNA level of TRKA. B. Western blotting analysis was used to detect the protein level of TRKA. C. Western blotting analysis was performed to determine the levels of E‐cadherin, N‐cadherin, α‐SMA, FN, and collagen I. ∗∗ p < 0.01, compared to the Si‐NC; ## p < 0.01, compared to the Si‐TRKA. [file CARJ-2026-6614209-s001.tif]
